# How relevant is social interaction in second language learning?

**DOI:** 10.3389/fnhum.2013.00550

**Published:** 2013-09-03

**Authors:** Laura Verga, Sonja A. Kotz

**Affiliations:** ^1^Department of Neuropsychology, Research group Subcortical Contributions to Comprehension, Max Planck Institute for Human Cognitive and Brain SciencesLeipzig, Germany; ^2^School of Psychological Sciences, The University of ManchesterManchester, UK

**Keywords:** language, learning, social interaction, communication, joint attention

## Abstract

Verbal language is the most widespread mode of human communication, and an intrinsically social activity. This claim is strengthened by evidence emerging from different fields, which clearly indicates that social interaction influences human communication, and more specifically, language learning. Indeed, research conducted with infants and children shows that interaction with a caregiver is necessary to acquire language. Further evidence on the influence of sociality on language comes from social and linguistic pathologies, in which deficits in social and linguistic abilities are tightly intertwined, as is the case for Autism, for example. However, studies on adult second language (L2) learning have been mostly focused on individualistic approaches, partly because of methodological constraints, especially of imaging methods. The question as to whether social interaction should be considered as a critical factor impacting upon adult language learning still remains underspecified. Here, we review evidence in support of the view that sociality plays a significant role in communication and language learning, in an attempt to emphasize factors that could facilitate this process in adult language learning. We suggest that sociality should be considered as a potentially influential factor in adult language learning and that future studies in this domain should explicitly target this factor.

## Introduction

In his book “Pragmatics of human communication” (1967), the psychologist and philosopher Paul Watzlawic stated that it is impossible not to communicate (Watzlawick et al., 1967). Indeed, in his view, every behavior is a form of communication intended to convey a message from a sender to a receiver (Shannon and Weaver, 1963). The interaction between partners crucially defines a communicative intention: while a sender and a receiver do not have to be present at the same time or in the same place, there is no communication without one of the two partners. The interactive nature of this process is reflected by the word “communication”, meaning “share with someone”, “let someone know” (from the Latin *cum*—with—and *munire*—to bind together). However, the study of the most widespread vehicle of human communication, language, has so far suffered from an individualistic approach. Here, we review recent findings bridging social cognition and communication by highlighting evidence that points towards the necessity to consider the impact of social interaction when investigating second language (L2) learning.

## Human communication and the role of social interaction

Human language is one of the most complex codes used to communicate between individuals. In its verbal form it is based on a small subset of sounds that can be combined in a potentially infinite number of bigger elements (words, phrases, sentences). The complexity of this code is further increased by the fact that human communication entails much more than the simple coding or decoding of linguistic utterances: For a communicative act to be effective, it is necessary for both the sender and receiver to understand the intentional state of a partner (Newman-Norlund et al., 2009; De Ruiter et al., 2010), an ability termed Theory of Mind (ToM) or mentalizing (Frith and Frith, 2006). The processes subtending ToM can be triggered by different contextual cues as long as they come from an agent (Frith and Frith, 2006); their function is to facilitate predictions about the others’ behavior via both verbal (Carruthers, 2002) and non-verbal (Noordzij et al., 2009; Willems et al., 2010) communication. An example of the latter case is reported in severe aphasic patients: although virtually unable to express themselves verbally, these patients are able to pass tests intended to specifically tackle their residual communicative abilities; for example, they are able to engage in intention recognition with a partner in a non verbal game requiring to signal the position of a specific target on a checkerboard (Willems and Varley, 2010; Willems et al., 2011). Another example comes from normally developing infants: although they have not yet developed verbal language, they are able to use the caregiver’s gaze direction as a cue to orient attention; this behavior requires a proto-mentalizing ability to infer the caregiver’s intention and represents one of the first communicative acts in children (Tomasello, 1995; Tomasello and Carpenter, 2007; Csibra and Gergely, 2009; see below). In adults mentalizing processes are activated by cues such as the identity of the person they are interacting with. In a recent study, Newmann-Norlund and colleagues demonstrated that in a non-verbal communicative task, adult participants adapted their communicative behavior to the presumed cognitive abilities of the partner. In the employed task participants had to communicate to a partner the spatial location of a target on a checkerboard by moving a token to the position of the target; they were told that the partner could either be an adult or a child. When they were prone to believe they were interacting with a child, participants spent more time moving the cursor, thus emphasizing a crucial element of communication such as the target location (Newman-Norlund et al., 2009). When the partner is a peer, adults still adapt their behavior; in most of the cases, this adaptation is reciprocal and results in behavioral resemblance between the partners. For example, pairs of adults tend to coordinate their body postures and gaze patterns during conversation, even without being aware of it (Shockley et al., 2007, 2009), and reduce the variability of their actions to better synchronize with each other (Vesper et al., 2011, 2012). Another example is the tendency to share feelings and emotions of others, often leading to the mimicry of an observed emotion (de Vignemont and Singer, 2006; Singer, 2006). An immediate evolutionary advantage of these phenomena is to facilitate learning mechanisms based on observation and imitation (Frith and Frith, 2012). However, how do these coordinative and imitative phenomena influence language? First of all, effective communication is based on the ability to know when it is the right moment to speak. This turn taking ability relies on general coordinative rules, both on the side of motor coordination (Shockley et al., 2009), and on the side of conversation. For example, you do not want your partner to wait forever for an answer, but you also do not want to speak while he is still speaking (“minimal gap, minimal overlap” rule, Stivers et al., 2009). Furthermore, aspects of a conversation, such as the speaking rate and the similarity of words spoken in a dyad, also influence the coordinative pattern as demonstrated by Shockley et al. (2007). The authors showed that pairs of participants were maximally synchronized in their bodily movements when they were uttering the same words at the same time (Shockley et al., 2007). Even more importantly, imitative motor phenomena are influenced by the conceptual level of the conversation: for example, hand gestures in a conversation are likely to be imitated and repeated by the partners, but only if they make sense in the context of the speech (Mol et al., 2012).

Taken together, this evidence suggests that there is a two-way influence between social interaction and communication. However, the role played by social interaction has been greatly undervalued so far, especially in studies on language learning, even though this context represents a prototypical interactive communicative situation. In the following sections, we will first describe technical limitations that may have been responsible for such paucity in research; then we highlight evidence on the impact of social interaction on learning in clinical and non-clinical populations.

## Brain imaging in interacting individuals: issues and solutions

Probably one of the reasons why social interaction has not been considered as a factor in language learning studies until recently is the limitation that dual settings pose to imaging set-ups. Luckily, the influence of an interactive social approach has increased exponentially over the last decade (Knoblich and Sebanz, 2006, 2008; Galantucci and Sebanz, 2009; Schilbach et al., 2013), leading to an attempt to find new techniques and to create experimental situations tailored towards real-life situations often involving more than one person (Montague et al., 2002; Hasson et al., 2012). This effort has lead to the development of paradigms intended to specifically tackle social situations (Schippers et al., 2010; Anders et al., 2011), in which participants are often made to believe that they are interacting with someone. For example, pairs of participants may be required to take turns in the fMRI scanner while observing a video recording of the partner during meaningful gestural (Schippers et al., 2009, 2010; Redcay et al., 2010) or affective (Anders et al., 2011) communication, while they believe this interaction is happening in real time. These kinds of “fake” communicative situations have allowed researchers to observe *in-vivo* activations in brain areas involved in the ToM system. This is supported by a network encompassing the medial prefrontal cortex (mPFC), the posterior superior temporal sulcus (pSTS), the temporo-parietal junction (TPJ), and the temporal poles (TP) (Amodio and Frith, 2006; Frith and Frith, 2006; Saxe, 2006; Decety and Lamm, 2007; Newman-Norlund et al., 2007; Noordzij et al., 2009). Another system usually involved in “social” tasks is the human Mirror Neuron System (MNS). This system encompasses a fronto-parietal network of the ventral premotor cortex (vPMC), the inferior frontal gyrus (IFG), and the inferior parietal lobule (iPL) in its rostral portion (Rizzolatti and Craighero, 2004), and possibly other regions, including the dorsal premotor cortex (dPMC), the supplementary motor cortex (SMA), and the temporal lobe (Keysers and Gazzola, 2009). Important for the topic of this review, these “mirror” neurons deal with the decoding of an action goal not only when one is performing an action, but also when observing the same action being performed by someone else (Rizzolatti and Fabbri-Destro, 2008; Keysers and Gazzola, 2009). These neurons thus provide an interface between one’s own motor repertoires and others’ (Knoblich and Sebanz, 2006). This “goal-sharing” property supports the hypothesis that brain areas exhibiting mirror-like properties should be more active during joint action than during solitary actions (Newman-Norlund et al., 2007). Although these “fake” social interactive tasks allow this hypothesis to be indirectly tested, recent developments in neuroimaging have allowed the creation of new techniques to be applied to fMRI (Montague et al., 2002), EEG (Astolfi et al., 2010, 2011), and NIRS (Cui et al., 2012), enabling two (and sometimes more) people to be tested at the same time. These “hyper-scanning” techniques (Dumas et al., 2011) allow ecologically valid interactions to be studied in a number of tasks, which could then also be applied to interactive learning paradigms. The clear advantage is that they allow a direct comparison of processes happening in two brains at the same time, a comparison which could otherwise only be inferred. Thus, one could potentially observe both the effects of mentalizing (King-Casas et al., 2005; Astolfi et al., 2010; Saito et al., 2010; Cui et al., 2012) and of synchronization (Tognoli et al., 2007; Schippers et al., 2010) on brain activity in a real-time learning set-up. The use of hyper-scanning in these tasks demonstrates that not only the behavior of two interacting people is influenced by social interaction, but also their brain activation patterns. Indeed, synchronized EEG activity in frontal and central regions has been found in theta and delta oscillations of pairs of guitarists playing a melody together (Lindenberger et al., 2009); similarly, when pairs of participants are required to spontaneously imitate each others, their brain activity becomes synchronized in the alpha-mu band over right-centro-parietal regions (Dumas et al., 2010). Activity in this frequency band has been proposed to represent a neuromarker of human social coordination and, more specifically, has been linked to the human MNS (Tognoli et al., 2007). Saito et al. (2010) used fMRI hyper-scanning to scan two people at the same time while they were engaged in a real-time gaze exchange; that is to say, the pair were asked to direct one anothers’ attention to an object via eye movements. The authors found that the exchange of attention via eye gaze resulted in an inter-subject synchronization of the neural activity in the right IFG (Saito et al., 2010). Mentalizing and mirror systems thus seem to be recruited in social tasks (Uddin et al., 2007; Van Overwalle, 2008; Van Overwalle and Baetens, 2009; Ciaramidaro et al., 2013), but their activity is influenced by the presence of a partner. Thus, the question arises: what happens in the case of learning a new language? A first attempt to answer this question arises from a recent study by Jeong and colleagues; the authors suggest that when words in a novel language are learnt in a social situation (but not when they are learnt from a text), elicited brain activity (in the right supramarginal gyrus) is similar to the activity elicited by words in one’s mother tongue (Jeong et al., 2010). However, the social situation depicted in this study was represented by movie clips of a dialogue. Thus, the question remains: what happens in a natural (social) learning situation?

## Language learning and social interaction in children

As previously pointed out, the ability to socially interact emerges very early in life (Grossmann and Johnson, 2007), and is represented by a number of basic interactions that children in the first year of life are able to master, such as following the caregivers’ gaze, attracting her/his attention, and responding to her/his attentional requests. This set of abilities is usually grouped under the name “joint attention”, entailing an interaction between a child, the caregiver, and the focus of attention (an object) (Carpenter et al., 1998; Mundy et al., 2003; Mundy and Sigman, 2006; Mundy and Newell, 2007; Mundy and Jarrold, 2010). From a psychological point of view, the role of triadic attention ability during childhood is to create a common psychological ground shared between the infant and the caregiver, and relies on the formation of ToM in children (Tomasello, 1995). In this common space, adults act as experts and guide the children toward the relevant information that should be learnt, by using an effective signal such as eye gaze (Csibra and Gergely, 2009; De Jaegher et al., 2010). In this asymmetrical learning setting, children behavior is further facilitated by the fact that adults tend to adapt their communicative behavior by emphasizing crucial aspects of communication (for example, by spending more time on them; Newman-Norlund et al., 2009). Moreover, the interaction with the caregiver increases motivation, thus reinforcing a given behavior (Vrtička et al., 2008; Hari and Kujala, 2009; Syal and Finlay, 2011). This asymmetrical learning setting, in which knowledge is passed from parents to offspring, is not limited to humans and can be found, for example, in many bird species that use complex vocal codes to communicate (Kuhl, 2007; Hari and Kujala, 2009; Frith and Frith, 2012). However, ToM abilities underlying human communication seem to represent a unicum in nature. Indeed, even our closer animal relatives, the chimpanzees, do not have the human ability to really “share” intentionality: as an example, chimpanzees are perfectly able to follow the gaze of an interacting human, but they do not try to start joint attention, nor do they try to infer the referent of the gaze as human children do (Tomasello and Carpenter, 2007). This human ability to share intentionality and acquired knowledge with other humans has been proposed to be at the core of the evolution of verbal language (Tomasello, 1995; Pinker, 2010). A series of experiments conducted by Kuhl and colleagues aimed to investigate this possibility and to test the impact of social interaction on phonetic discrimination in children (Kuhl et al., 2003; Kuhl, 2007). Cohorts of American infants were exposed to native speakers of Mandarin Chinese and subsequently performed a phonetic discrimination task; the exposure either occurred via direct interaction or via pre-recorded video tapes. Interestingly, infants were able to learn different Mandarin phonemes when they were exposed to them by a real person, but not when the exposure was merely via a recording (Kuhl et al., 2003). There are two plausible explanations for this effect; first, a live human may attract more attention and increase motivation, as compared to a recording. Second, a real person can provide referential information, crucial for linking words and concepts (Waxman and Gelman, 2009). In particular, Kuhl and colleagues pointed out that joint attention towards an object being named can facilitate a child’s capacity for word segmentation (Kuhl et al., 2003). Similarly, results from Hirotani et al. (2009) suggest that joint attention helps to strengthen the association between a word and its referent, thus facilitating learning. These authors found that semantic integration, reflected in the N400 effect, seemed to be present when children learn new words in a joint attention condition but not in a non-joint-attention context. Although infant learning represents a particular case, vocabulary learning poses similar demands to both children—learning their first language (L1)—and to adults—when learning a new language. Thus, factors facilitating word learning in children could potentially impact adult learners in a similar way.

## The role of sociality in second language learning

Evidence thus accumulates to favor the view that the development of verbal language is, at least, supported by establishing common ground between a sender and a receiver. In turn, the events that take place in such common space are mostly dependent on the interaction between the partners (Mundy and Jarrold, 2010). However, a note of caution needs to be used when comparing language learning in children and in adults. Indeed, learning of a L2 can occur largely independent of the presence of another person, and is usually learnt via explicit formal training as compared to a L1, which is acquired effortlessly without explicit instructions (Abutalebi, 2008). Nevertheless, the case of word learning represents a link between language learning in infants and in adults. Indeed, words in a new language can be acquired incidentally (Nagy et al., 1987; Swanborn and De Glopper, 1999; Laufer and Hulstijn, 2001; Rodríguez-Fornells et al., 2009); new words encountered while reading a text can be easily learnt. In this situation an adult learner has to face the same problems as an infant, namely the indetermination of the referents: there are multiple words in a language and multiple possible referents in terms of meaning. However, how can the correct meaning be assigned to an unknown word? The easiest way to go about this problem is exemplified by associative learning, a procedure that concentrates on the statistical learning of the co-occurrence of data from speech and its context (Breitenstein et al., 2004; Whiting et al., 2007, 2008). The advantage of this procedure is that it poses low cognitive demands during training (Pulvermüller, 1999; Dobel et al., 2010) and is resistant to errors made during a phase of guessing (Carpenter et al., 2012). The underlying rationale is that once a word is heard in an utterance or seen in a sentence, a set of potential meanings can be inferred from the context, thus reducing the number of possible referents (Adelman et al., 2006). This way, novel word forms can be acquired and integrated in the lexicon relatively quickly and successfully. For instance, neural responses evoked after training are indistinguishable from those obtained in response to “old” words, as demonstrated in the disappearance or reduction of a N400 response (Mestres-Missé et al., 2007). The N400 component is a negative deflection starting 200–300 ms after the presentation of a word, and has been associated with semantic processing (Lau et al., 2008). Its disappearance in a learning paradigm thus possibly corresponds to establishing a link between a novel lexeme and conceptual information (Mestres-Missé et al., 2007; Dobel et al., 2010). The neural network supporting word learning involves regions of the semantic circuitry such as the left IFG (BA 45), the middle temporal gyrus (MTG, BA 21), the parahippocampal gyrus, and several subcortical structures (Mestres-Missé et al., 2008). Although, in adults, new vocabulary can be learnt independently of the presence of a partner, social interaction may increase the number of cues and referential information in much the same way as it does in infant learning (Kuhl, 2004, 2007, 2010). Indeed, the interaction between partners in conversation could lead L2 learners to focus on certain aspects of the context and certain words in speech (Yu and Ballard, 2007). The coordinative phenomena we describe above could play a role in this process, maximizing the efficiency of the conversation and consequently facilitating the focusing of attention: this proposal has been made for word learning in toddlers. Indeed, it has been shown that in toddler-adult dyads, the number of new words learnt by the toddlers is proportional to the quality of the synchronization during the interaction with the caregiver (Pereira et al., 2008). Again, it is important to note that the case of word learning is not dissimilar in adults and infants, and so one may expect facilitating factors (such as the focusing of attention driven by synchronization) to play a role in word learning for both adults and children. Indeed, although it is possible to learn a new language alone, adults often learn a new language in social contexts, most commonly in a teacher—learner setting, a setting which requires interaction with a partner as well as sophisticated reading of a speaker’s intentions (Bloom, 2002; Mestres-Missé et al., 2007, 2008). Thus, the necessity to consider sociality as a factor in L2 studies seems striking, as further suggested by the evidence that when new words are encoded in a social context, but not when they are learnt by translation, the pattern of activation in the retrieval phase is similar to the one observed for L1 words (Jeong et al., 2010).

## Learning and social cognition in pathologies

Learning new words, or re-learning words that have been forgotten, is the goal not only for infants and L2 learners, but also for pathological populations, including, for example people suffering from autism, dementias, or aphasia. In these pathologies, the role of social interaction is becoming increasingly acknowledged as a crucial variable for therapeutic outcome success. Communicative deficits in autism spectrum disorders have been frequently attributed to higher cognitive processing impairments, and especially to ToM deficits (Baron-Cohen et al., 1985). However, more recent evidence indicates that lower level processes may also be affected. For instance, recent findings suggest that autistic children display low-level difficulties in temporal processing, including impaired timing and deficits in the perceived duration of an event, which can in turn influence the perception of relevant social cues such as eye gaze (Allman, 2011; Allman et al., 2011; Falter and Noreika, 2011; Falter et al., 2012). The fact that ToM and timing abilities may be crucial for language, even in a population who display impaired ToM, comes from the discovery that autistic children improve their language abilities after a treatment focusing on the optimization of their joint attention capacities (Kasari et al., 2008).

Similarly, social interaction plays a role in language re-learning in aphasia. A paradigmatic example of this claim comes from a specific form of therapy for severe aphasic patients based on music, namely Melodic Intonation Therapy (Norton et al., 2009). This approach uses musical and sensory stimulation in order to improve the speech production of the aphasic patient and is centered on the role of the therapist. Although the beneficial effect of the therapy has been traditionally attributed to the effect of music tout-court, recent evidence challenges this perspective and suggests that rhythm (and not necessarily melody) holds the key to understanding the impact of music therapy (Stahl et al., 2011). Considering that music therapy is therapist-centered, this result well fits a joint-action explanation: rhythm is defined by the coordinated action between a therapist and a patient. This strongly influences timing and its variability of the single individual in the interaction. Future investigations should attempt to disentangle the role played by joint action dynamics from those played by the timing of the interaction, per se.

## Concluding remarks

In conclusion, the role of social interaction in language learning has, thus far, been widely overlooked, partly because of technical constraints posed by interactive settings in imaging studies. We propose that further studies on language learning in adults should further explore the powerful impact of social interaction. This necessity comes from at least four lines of research: first, language use intended as communication is an interactive phenomenon, relying on the ability of the partners to infer the others’ mental states and to coordinate with each other in successful turn-taking. Second, in infants, joint attention with a caregiver provides additional contextual cues driving attention and motivation that can help to disambiguate the meaning of a new word (or stimulus); analogously, contextual learning represents one of the easiest ways for late learners to acquire new words and can thus be influenced in a similar way by social interaction. Third, and related to the second, the investigation of interactive language learning resembles a natural learning situation involving a teacher and a student. Fourth, the role of sociality is starting to emerge as a valid explanatory variable in the context of word learning in pathological populations.

## Conflict of interest statement

The authors declare that the research was conducted in the absence of any commercial or financial relationships that could be construed as a potential conflict of interest.
